# Progress in Medicine

**Published:** 1896-12-12

**Authors:** 


### Progress in Medicine.

### DISEASES OF THE NERVOUS SYSTEM.

*(Continued from page 166.)*

**Tic Douloureux.**—*Treatment:* In the treatment of the cases narrated by Ewart[17] special regard was directed to the following indications: (1) The sedative; (2) the restorative; (3) the alterative; and (4) the tonic.

1. Sleep being the first essential, and the basis of the entire treatment, it is necessary that the narcotic prescribed should be sufficiently strong to procure prolonged slumber. Chloral and morphine combined will usually prove effectual.

2. The restorative measures include mental and bodily rest and suitable alimentation. Rest in bed is indicated in all severe cases till remedies have taken effect; and the success of treatment may largely depend upon this means of economising nerve energy. Alimentation is the second essential, but needs to be adapted to the irritable state which prevails at this stage. The object of the treatment is to raise the nutrition without overloading the blood with nitrogenous waste. Thus, a modified vegetarianism, including a liberal supply of the vegetable foods and a moderation of the nitrogenous, is generally indicated. Vegetarianism itself deserves a full trial in the worst cases before resorting to operation, and it is said to have been attended with most favourable results, though Dr. Gower refers to the opposite experience in a case where the neuralgia began coincidentally with the adoption of the vegetarian plan. Vegetable food was liberally, but not exclusively, supplied in the present cases. Alcohol is to be avoided. Sufferers are well aware that the relief it affords is purely temporary; and it is regarded by the writer as decidedly harmful in promoting a liability to the attacks. Whilst this prevails it should be absolutely forbidden; and, subsequently, total abstention is the safer course, and should be the rule in the gouty cases. Nevertheless, in enfeebled and underfed subjects, stout, or even port wine, may be allowed as a temporary help to nutrition.

Dyspepsia, due to gastro-hepatic catarrh, may have to be dealt with as a preliminary; and, in a mild case of this kind, the neuralgia may rapidly subside under treatment directed to the stomach. The state of the teeth and of the gums also calls for every attention, though, as regards the teeth, more than the needful has often been done before the patient comes under treatment.

3. The alterative treatment may, in genuinely gouty cases, be specially directed to the gout, with careful avoidance, however, of any depressing agents. In the generality of cases a modified treatment, favouring absorption as well as glandular activity (particularly that of the liver), will meet the gouty as well as the general indication. The salicylates, the benzoates, sulphur, chloride of ammonium, and taraxacum are available, but none of them probably equal in efficacy the salts of iodine and of mercury, and particularly their combination in the proportion of twenty to thirty minims of the solution of the perchloride of mercury, and of six to ten grains or more of the iodide of potassium. The iodide may be tried alone, and should then be given in sufficient doses. Its action seems to be promoted by the addition of tincture of iodine in doses of fifteen to thirty minims. The full measure of relief, however, was not obtained in the two cases where the drugs were administered in instalments until mercury was added to the iodides.

4. The tonic measures are less dependent on drugs than on hygiene, and, above all, on muscular exercise. Muscular exercise should at first be passive.

Ewart remarks, in conclusion, that under the treatment described by him all the cases improved, and lost the pain for a time, although it had existed for prolonged periods. Yet these were cases sent to the surgeon for the express purpose of operation because medical treatment had failed or was regarded as useless; and they might, on that showing, have been operated forthwith as intractable cases. In all of them the evil day had been postponed; in some, perhaps, indefinitely. A radical cure, one which would compare with the cure of a neoplasm by the removal of the growth, is not to be expected; if the affection is largely functional a renewal of its occasioning causes must revive it; but the temporary relief afforded is in itself evidence that the ailment is not necessarily a progressive one, and that it may be amenable to treatment, even when of old date. If the symptoms can be checked once, may they not be checked again, and as often as there may occur any sign of a relapse? This question is partly answered by some of the cases, but should be put to a wider test. Lastly, it is still a question whether the series given has been a specially favoured one, and whether cases may occur of a still more severe type, upon which no impression can be made by medical treatment.

In a case recorded by Tourette[18] he adopted a line of treatment which was one recommended by Trousseau, which Tourette had frequently seen employed successfully by Charcot. It is based principally on the administration of extract of opium in large and increasing doses. The initial dose that Tourette generally recommends is six, eight, or ten centigrammes, administered at equal intervals during the twenty-four hours. We may increase the dose until the pain ceases, but the pain required for this varies in each case. In one instance relief may be obtained from a dose of twenty centigrammes, or even less, while another patient may require double this dose. Apart from absolute intolerance to the drug even in small doses, which is exceptional, various ill effects may supervene which interfere with the treatment. Peculiarly enough it is less drowsiness and general numbness that one has to dread in such cases than general disturbances.

When the painful symptoms have disappeared, Tourette advises to keep up the maximum dose for a few days, and then diminish progressively; but should the neuralgia threaten to recur, increase the number again until this tendency has been overcome.

**Neurasthenia.**—Much has been written by a few observers on the all-important subject of neurasthenia, the disease *par excellence* of *fin de siècle* high pressure in living. Yet how little the full import of this condition is realised by practitioners generally. How many cases of complete collapse, of irrevocable damage to the higher nerve centres, and of insanity might be arrested in time were we only to keep ourselves alive to the earlier clinical indications of the neurasthenic state! Edgar Ivatts,[19] in writing on "the necessity of economy in nerve expenditure," draws attention to the fact pointed out by Herbert Spencer in his "Principles of Psychology," that there are in the nerve cell, as in other cells, two opposed processes in continual operation, termed by the psychologists anabolism and katabolism. During periods of great activity the katabolic changes are greatly in excess of the anabolic, and proceed at their expense. There is a point of extreme divergence on the katabolic side, an approach to which is compatible with health. This point no doubt varies with different individuals according to their constitutional strength, and also, no doubt, in the same individual somewhat according to the condition of the health at the time. The bicyclist goes of his will until he feels too fagged to go farther, and wisely gets off. The sensible man of business comes home tired, refuses to go out again, and goes to bed early. The busy, overworked housewife gets extra help, or her husband sends her off for a holiday to recuperate; and then a working equilibrium is maintained. But if, under strain of business, or from other causes the natural checks or deterrents to excessive expenditure are disregarded, what happens? The supreme inhibitory force called will, or self-control, is first exhausted, and then follows what might be called "hyper-katabolism," or exalted action of the subordinate nervous centres, with which every physician is familiar, and which is the first indication and sign of threatened disintegration. A person in such a condition has for a time an unusual sense of well-being and elation, with increased capacity for work. His ideas flow rapidly and without effort, and his conversation is unusually fluent. Work that at other times would seem a great deal to accomplish seems easy of accomplishment. A prospect that was previously not very inviting now seems rosy. Such a condition is of course common to ordinary healthy people, but there are indications which show that the normal equilibrium has been upset. The true relations of such persons to their surroundings is to some extent lost, as in a person under the influence of alcohol, and they are borne along involuntarily in the current of their own ideas. There is sometimes a wild restless look in his eyes, and there is exalted action of the motor centres, with some loss of muscular sensibility. Such persons find a difficulty in sitting still, and the ordinary co-ordinate movements are exaggerated, so that they move rapidly, and continue walking and running upstairs without feeling tired. When sitting down, a constant shifting of position, or agitated chair rocking—a general fidgetiness—may be noticed. Their only complaint at first is a disagreeable feeling in the epigastrium, probably due to hyperæsthesia. It will be found on inquiry that this display of energy is not being compensated for with food and rest. On the contrary, it will found that the usual sleeping hours are reduced to three or four, or very commonly there is complete insomnia. Such a condition precedes many cases of mental disintegration, and is familiar in a more exaggerated form as the early stage of acute mania and general paralysis. If at this stage the patient comes under a physician and can be persuaded to alter his or her mode of living by adopting the only rational measures that lead to recovery (rest and food), the end may be averted. Unfortunately, this advice is too often disregarded, and as the inevitable reaction sets in, recourse is had to the various so-called "nerve tonics"—too often alcohol, morphine, or cocaine. These are no better than spurs to a jaded horse. After a certain time the reserve is exhausted, and there is a progressive depression of the nervous system, spreading from the higher centres downwards. All work becomes impossible; a settled melancholy succeeds the previous exaltation; the presence of intimate friends, and even of nearest relatives, becomes a burden; various forms of emotional weakness, with hysterical disturbances, manifest themselves. In a large proportion of cases, as the result of extended rest, the nominal equilibrium is re-established, and the patient returns to his or her usual occupation. This means that a considerable rest has to be taken, extending in some cases to a couple of years or more, but there are some unfortunate cases in which the normal equilibrium is never re-established, and a permanent unstable condition of the nervous system results. The observations of Ivatt's are worthy of careful consideration.

[17] Loc. cit. [18] Loc. cit. [19] New York Med. Record, June 13, 1896.

